# An integrated epigenomic analysis for type 2 diabetes susceptibility loci in monozygotic twins

**DOI:** 10.1038/ncomms6719

**Published:** 2014-12-12

**Authors:** Wei Yuan, Yudong Xia, Christopher G. Bell, Idil Yet, Teresa Ferreira, Kirsten J. Ward, Fei Gao, A. Katrina Loomis, Craig L. Hyde, Honglong Wu, Hanlin Lu, Yuan Liu, Kerrin S. Small, Ana Viñuela, Andrew P. Morris, María Berdasco, Manel Esteller, M. Julia Brosnan, Panos Deloukas, Mark I. McCarthy, Sally L. John, Jordana T. Bell, Jun Wang, Tim D. Spector

**Affiliations:** 1 Department of Twin Research & Genetic Epidemiology, King's College London, London SE1 7EH, UK; 2 BGI-Shenzhen, Shenzhen 518083, China; 3 Wellcome Trust Centre for Human Genetics, University of Oxford, Oxford, OX3 7BN, UK; 4 Pfizer Worldwide Research and Development, Groton, Connecticut 06340, USA; 5 Department of Biostatistics, University of Liverpool, Liverpool L69 3GA, UK; 6 Cancer Epigenetics and Biology Program (PEBC), Hospital Duran i Reynals, Barcelona 08908, Spain; 7 Department of Physiological Sciences II, School of Medicine, University of Barcelona, Barcelona, Catalonia 08907, Spain; 8 Institucio Catalana de Recerca i Estudis Avançats (ICREA), Barcelona, Catalonia 08010, Spain; 9 Pfizer Worldwide Research and Development, Cambridge, Massachusetts 02139, USA; 10 William Harvey Research Institute, Barts and The London School of Medicine and Dentistry, Queen Mary University of London, London EC1M 6BQ, UK; 11 King Abdulaziz University, Jeddah 22254, Saudi Arabia; 12 Oxford Centre for Diabetes, Endocrinology and Metabolism, University of Oxford, oxford OX3 7LE, UK; 13 Oxford NIHR Biomedical Research Centre, Churchill Hospital, Oxford OX3 7LE, UK; 14 Department of Biology, University of Copenhagen, Copenhagen DK-2200, Denmark; 15 The Novo Nordisk Foundation Center for Basic Metabolic Research, University of Copenhagen, Copenhagen DK-2200, Denmark

## Abstract

DNA methylation has a great potential for understanding the aetiology of common complex traits such as Type 2 diabetes (T2D). Here we perform genome-wide methylated DNA immunoprecipitation sequencing (MeDIP-seq) in whole-blood-derived DNA from 27 monozygotic twin pairs and follow up results with replication and integrated omics analyses. We identify predominately hypermethylated T2D-related differentially methylated regions (DMRs) and replicate the top signals in 42 unrelated T2D cases and 221 controls. The strongest signal is in the promoter of the *MALT1* gene, involved in insulin and glycaemic pathways, and related to taurocholate levels in blood. Integrating the DNA methylome findings with T2D GWAS meta-analysis results reveals a strong enrichment for DMRs in T2D-susceptibility loci. We also detect signals specific to T2D-discordant twins in the *GPR61* and *PRKCB* genes. These replicated T2D associations reflect both likely causal and consequential pathways of the disease. The analysis indicates how an integrated genomics and epigenomics approach, utilizing an MZ twin design, can provide pathogenic insights as well as potential drug targets and biomarkers for T2D and other complex traits.

## Abstract (web summary)

Type 2 diabetes (T2D) is a highly heterogeneous disease with a strong genetic component. Here the authors examine genome-wide methylation patterns in T2D-discordant, T2D-concordant and healthy concordant monozygotic twin pairs, and identify DNA methylation signals that may represent new biomarkers or drug targets for T2D.

## Graphical abstract

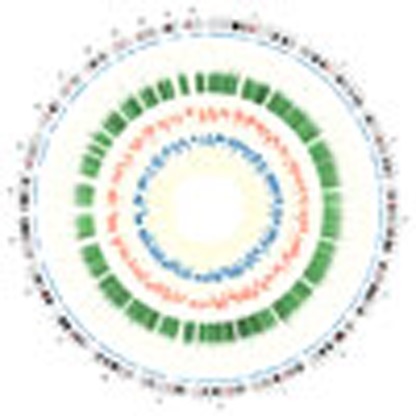

Type 2 diabetes (T2D) is a highly heterogeneous disease caused via a combination of genetic susceptibility and environmental exposures. Recent genome-wide association studies (GWAS) have identified at least 65 T2D loci, which explain only ~6% of disease susceptibility variance^1^. Part of the variance in T2D could also be explained by epigenetic effects, such as differences in DNA methylation^2^,^3^. Finding disease-associated DNA methylation variation will provide insight into novel molecular disease mechanisms, may help to predict disease status and potentially generate novel treatment methods. The identification of these genome-wide differentially methylated regions (DMRs) in association with complex phenotypes through epigenome wide association studies^3^,^4^, is substantially improved by a powerful disease-discordant identical twin model^5^. Previous studies in psychiatric disease and psoriasis using this twin model with Illumina HumanMethylation27K arrays have provided suggestive, but unreplicated evidence of associations^6^,^7^, and more recent analyses with the Illumina HumanMethylation450k array in unrelated subjects have identified significant effects in autoimmune disease, within the HLA region^8^.

In this study, we use high-throughput methylation immunoprecipitation sequencing (methylated DNA immunoprecipitation sequencing, MeDIP-seq) to obtain whole-blood DNA methylation profiles. MeDIP-seq enables a cost-effective genome-wide DNA methylome characterization and provides access into moderately dense CpG regions of the genome, such as CpG island ‘shores’ (refs 9, 10) and repetitive elements with potential regulatory effects^11^. We compare genome-wide DNA methylation profiles in T2D to identify DMRs in the total set of 27 discordant and concordant pairs of monozygotic (MZ) adult twins using a mixed effect model. We follow these up with analysis restricted to the 17 T2D-discordant MZ twins to identify genetically independent DMRs (giDMRs) with a paired model. Importantly, we replicate our results in an independent sample of 263 unrelated cases (42) and controls (221).

## Results

### DNA methylome analysis in MZ twins

**Figure 1:**
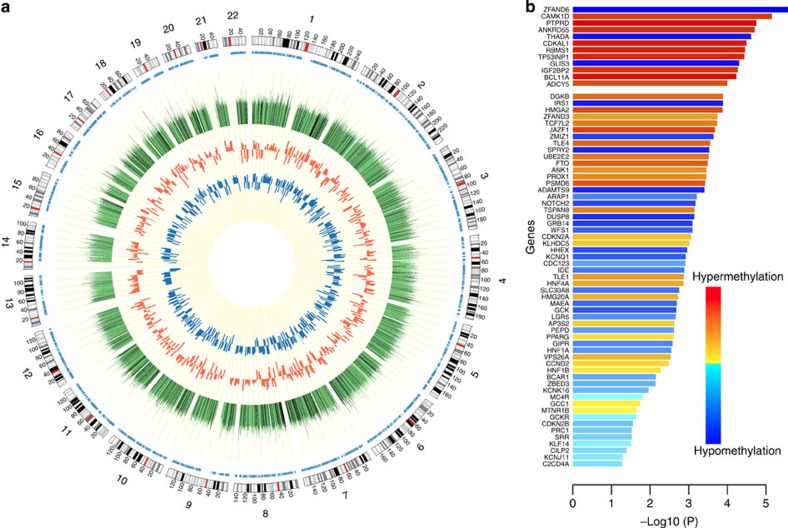
T2D-DMR genome-wide distribution. (**a**) Circos plot of the T2D-DMR distribution. Green track indicates the association of DNA methylation in each bin with T2D status (−log10 (linear mixed effect model p value)). Labelled in black are the 1,355 replicated T2D-DMRs. The middle circle indicates the effect of the most significant replicated DMRs per 10 Mbp in MZ discovery samples. The inner circle indicates the effect of the DMRs in the replication set. (**b**) The association between DNA methylation and T2D status at T2D genes nearest to T2D-GWAS loci. The genes are ranked by the significance of the T2D-DMR effects, such that the top 12 genes are within the set of 68 genome-wide FDR=25% T2D-DMR genes. The bar colour shows the direction of methylation effects from hypermethylation to hypomethylation.

MeDIP-seq data were generated at ~30 million paired-end reads of length 50 bp per individual, and mapped to the human genome using Novoalign^12^. We quantified relative levels of DNA methylation in overlapping bins of size 500 bp (sliding window of 250 bp) using MEDIPS^13^. We assessed evidence of DMRs in T2D (T2D-DMRs) in the entire sample of 27 MZ twin pairs, which consisted of 17 T2D discordant, 3 T2D concordant and 7 healthy control concordant twin pairs (Supplementary Data 1). In this exploratory stage, we identified one disease-associated DMR at a FDR level of 5%, 31 DMRs at 10% and 4,545 DMRs at 25% using a linear mixed effects model. Taking the FDR 25% cutoff as our suggestive T2D-DMR set, approximately two-thirds of the observed suggestive T2D-DMRs are hypermethylated in cases (Fig. 1). Using annotations to 53,631 autosomal Ensembl genes (19,816 protein coding, 22,013 non-coding, 11,802 pseudogenes) 1,535 suggestive T2D-DMRs (33.7%) are located in the extended promoter region (within 20 kb upstream of the gene including potential distal promoter and nearby regulatory elements^14^) and 2,494 (54.8%) are found to reside within the gene body. In total, 3,597 genes are annotated to contain at least one suggestive T2D-DMR within the gene body or promoter region (Supplementary Data 2). Exactly 0.8% of the T2D-DMRs are located within 100 bp of the transcription start site (TSS), which is 34% more than that expected by chance (comparing observed data to randomly permuted TSS locations within chromosome for 20 permutations, *P*<0.05). We also assess the overlap between the suggestive T2D-DMRs with the most variable DNA methylome regions (totalling 492 Mb) detected by whole-genome bisulfite sequencing from 42 data sets across 30 human cell and tissue types defined by Ziller *et al*.^15^ Altogether, 867 T2D-DMRs (19%) co-localize with DNA methylation variation loci, which translates to a 1.1-fold enrichment (Fisher’s test *P*=0.003).

To further validate our MeDIP-seq methylome analysis, we also assess the highly-replicated DNA methylation association with smoking status in the genes *ALPPL2* (2q37.1 region)*, AHRR* and *F2RL3* within our data set. We selected previously identified smoking-associated 450k CpG sites in these genes, and extracted MeDIP-seq bins that overlap the smoking-associated CpG site. We find significant associations between smoking status and methylation levels at CpG sites in the *ALPPL2* and *AHRR* genes (Bonferroni corrected *P*<0.05) and borderline uncorrected significance for the *F2RL3* gene. These findings are reassuringly consistent with recent discoveries, despite the small numbers of smokers in our sample (11 out of 54) and the use of different technologies.

### T2D-DMRs validation and replication

To validate our MeDIP-seq results with another platform, we targeted the top 24 DMRs from the more stringent FDR 10% set and 7 other suggestive T2D-DMRs in the same set of samples using the Illumina HumanMethylation450K Bead Chip (Supplementary Data 3). Of these 31 DMRs, 20 DMRs have representative 450k probes for the validation test. Of these 20 DMRs, 12 DMRs are validated with the same direction of effect at nominal significance (linear mixed effect model *P*<0.05), and a further 4 DMRs show the same direction of effect, but do not reach nominal significance.

We then attempted replication of all 4,545 suggestive T2D-DMRs in an independent sample of 42 unrelated T2D cases and 221 UK controls, matched for age, body mass index (BMI) and sex with single-end MeDIP-sequencing profiles. Of the 4,545 DMRs, 3,939 (87%) show the same direction of association and 1,355 (30%) replicate with the same direction of association and with nominal significance (linear regression *P*<0.05).

### MALT1 methylation associates with T2D

**Figure 2:**
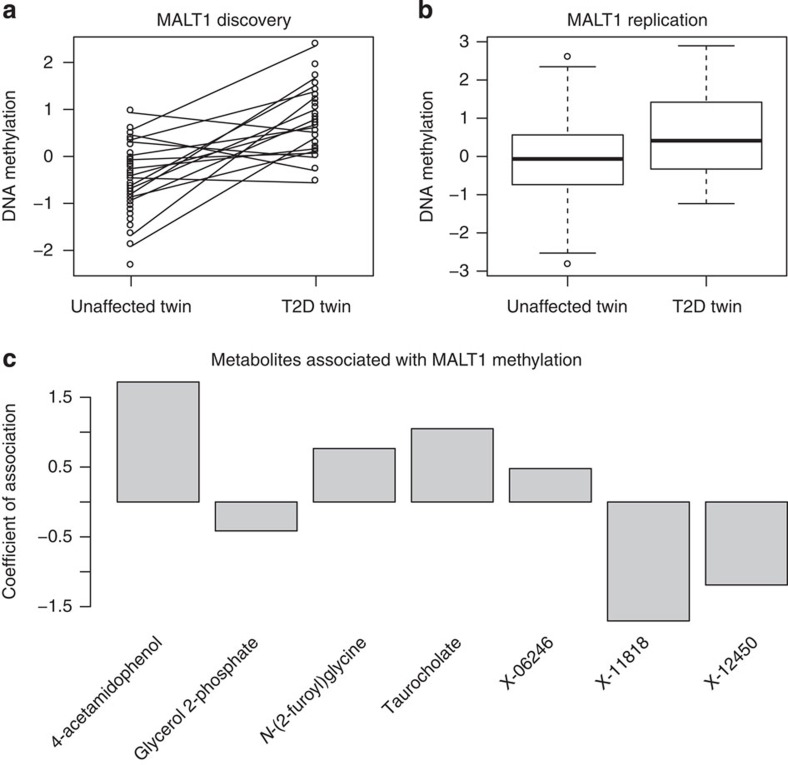
DNA methylation and metabolomics profiles at MALT1. Normalized RPM levels in T2D cases and unaffected controls in the discovery (**a**) and replication (**b**) data sets. (**a**) Solid black lines link each pair of 17 T2D-discordant twins. (**b**) Association profiles in the replication set of 263 individuals with s.e. (**c**) Significant metabolites associated with levels of DNA methylation in the data set, and direction of association.

The top ranked T2D-DMR (chr18:56336501–56337000, *P*=9.95 × 10^−10^, *β*=0.08, FDR 5%; replication *P*=2 × 10^−3^) overlaps another T2D-DMR (chr18:56336751–56337250, *P*=6 × 10^−5^, FDR 23%, *β*=0.1, replication *P*=5 × 10^−4^, Fig. 2). These DMRs are located in a 2kb region upstream of the TSS within the 5′ promoter (as defined by ChromHMM analyses in GM12878 (ref. 16)) of the MALT1 gene (mucosa-associated lymphoid tissue lymphoma translocation protein 1). *MALT1* is a signalling protein with a key role in antigen receptor-mediated lymphocyte activation through the nuclear factor-κB pathway, important in the development and function of B and T cells^17^, as well as energy and insulin pathways^18^. We explored genetic contributions to *MALT1* regulation, but found no evidence that genetic variants are associated with either *MALT1* methylation (in the replication sample) or expression in adipose, lymphoblastoid cell lines , skin tissue (using the MuTHER gene expression data set from the same twin population^19^). Furthermore, whole-blood DNA methylation in *MALT1* did not associate with its gene expression in the three tested tissues, although the gene has previously been shown to be expressed in whole blood^20^.

To explore a potential functional influence of the *MALT1* DMR on T2D, we correlated the top DMR’s (chr18: 56336501–56337000) methylation patterns with 503 fasting blood metabolites profiled by mass spectrometry^21^. Seven metabolites consistently associate with *MALT1* methylation in both the discovery and replication samples (meta-analysis Bonferroni correction *P*<0.05; Supplementary Data 4). Among these is taurocholate, which has been associated with increasing L cell and insulin secretion as well as a decrease in blood glucose and food intake in obese type 2 diabetic volunteers^22^.

### Enrichment in T2D GWAS loci and imprinted genes

We next integrated our DMR findings with GWAS meta-analysis results^1^ and found significant overlap of T2D-DMRs and GWAS loci using three approaches. First, we compare the most significant single nucleotide polymorphisms (SNPs) within each of the 65 T2D GWAS loci (50 kb either side of the SNP) with our 4,545 suggestive T2D-DMRs (FDR 25%), and observe a significant overlap of genomic regions (Fisher’s test *P*=0.0004). Second, we compare the 65 genes closest to the published GWAS T2D loci with our 3,597 T2D-DMR genes, and find highly significant overlap of genes (Fisher’s test *P*=0.004). Third, the ranking of DMR *p* values within the GWAS T2D loci in the genome is significantly higher than that for sets of randomly sampled Ensembl genes that had MeDIP-seq coverage (10,000 draws Wilcoxon ranking test *P*<0.0001; Supplementary Data 5). In total, 12 of the 65 reported T2D GWAS loci are significantly differentially methylated (FDR 25%), and these include well-documented T2D loci such as those near *IGF2BP2* (Insulin-like growth factor 2 mRNA-binding protein 2) and *THADA* (thyroid adenoma-associated gene). The DNA methylation levels at these 12 T2D GWAS loci clustered the individuals in our study into two clear case–control groups (Supplementary Fig.1a,b). As a control, and a check for bias and specificity, we found no enrichment of GWAS genes related to other diseases and phenotypes, such as psoriasis, Crohn's disease and hair colour (Supplementary Data 6). The significant overlap between T2D-DMR genes and T2D GWAS loci suggests that DNA methylation or related chromatin structure alterations of many T2D-DMR genes could be causally related to T2D status and in theory potentially reversible, which therapeutically could have major clinical promise.

We further cross referenced our top 3,597 genes nearest to the 4,545 suggestive T2D-DMRs (FDR=25%) with well-established imprinted loci from The Genomic Imprinting Website^23^. We examined imprinting regions because: (1) previously published studies have suggested a role for parent-of-origin effects in T2D^24^,^25^; (2) obesity, being a risk factor for T2D, is a common phenotype in syndromic imprinting disorders, and has therefore implicated aberrant imprinting in common obesity susceptibility; (3) the developmental hypotheses that have been proposed in these diseases^25^,^26^,^27^,^28^. T2D-DMRs overlap with 191 potentially imprinted genes with a threefold enrichment (Fisher’s test *P*=0.001), and furthermore show a twofold enrichment with 83 confirmed imprinted genes (Fisher’s test *P*=0.1). This includes genes previously linked to diabetes and obesity, for example *PRDM16* (refs 29, 30). We also find a higher ranking of DMR *p* values for the imprinted loci than for randomly sampled genes that have MeDIP-seq mapping coverage (*P*=8 × 10^−4^; Supplementary Data 7).

### DMRs associate with genetic variants

To further investigate the genetic contribution to the T2D-DMRs, we attempted to identify methylation quantitative trait loci (mQTL), and thus associate methylation levels with genotype variation. For *cis* mQTL, we tested common SNPs within 50 kb of the T2D-DMRs and found an enrichment for genetic effects, suggesting that a number of the disease DMRs have a genetic contribution. Altogether, 397 out of the 4,545 T2D-DMRs possess a *cis* mQTL (linear regression *P*=8.6 × 10^−7^, FDR=5%). For example, SNP rs10495903 associates with a DMR 8 kb from the 5′ end of *THADA* (chr2:43831251–43831750, *P*=6.7 × 10^−10^, *β*=−11.4) and SNP rs2086675 associates with an intragenic *ANKRD55 (Ankyrin Repeat Domain 55)* DMR (chr5:55464001–55464500; linear regression *P*=6.4 × 10^−7^, *β*=7.0). We further assessed genetic *trans* associations for 757 replicated T2D-DMRs (replication linear regression *P*<0.01) in the replication samples. Of these 757 T2D-DMRs, 29 (3.8%) have *trans* mQTL (linear regression *P*=2.4 × 10^−8^, FDR=5%), but none show significant evidence that methylation is the mediator of the genetic effect on disease using the causal inference test^31^.

### Discovery of giDMRs

Using our monozygotic discordant twin pair model, we are also able to search for epigenetic differences within genetically identical twins, and we name these regions ‘genetically independent DMRs (giDMRs). Unlike T2D-DMRs identified from the linear model, which reflect both genetic and environmental effects on T2D, giDMRs likely represent pure environmental effects, stochastic mechanisms or consequences of the disease itself. We find 2 (*t*-test *P*=3 × 10^−7^, FDR=10%) and 914 (*t*-test *P*=1 × 10^−4^, FDR=25%) T2D giDMRs within the 17 discordant MZ twin pairs, which overlap 890 genes in total (including regions up to 20 kb upstream of the gene, Supplementary Data 8,9). The giDMR genes are significantly enriched in the Wnt signalling pathway, insulin signalling pathway and synaptic vesicle cycle (FDR=0.001, 0.003 and 0.004, respectively Supplementary Data 10). We tested the T2D-methylation association of the top 20 gene-related giDMRs in the replication data set of 263 unrelated cases and controls, and find that three regions validate with same direction of effect at nominal significance (linear regression *P*<0.05), and a further eight show the same direction of change. We also observe that three of the top four DMRs showed the same trend of association in skeletal muscle in another unrelated cohort of 30 cases and controls profiled with the Illumina 450k array. Altogether, 175 giDMRs (which mapped to 109 genes) overlap the 4,545 FDR 25% T2D-DMRs from the linear mixed model analyses. The *MALT1* DMRs do not pass the FDR 25% giDMR threshold, but show modest giDMR effects consistent with the direction of T2D-DMR association in the primary linear model, suggesting that they were primarily independent of genetic influences (chr18:56336501–56337000, *P*=0.001). The top giDMR, which is also identified in the primary linear model analyses (discovery *P*=3.78 × 10^−6^ and replication *P*=0.01), is in the promoter of the *GPR61* (G-protein coupled receptor 61) gene. Knockout studies of *GPR61* in mice exhibit hyperphagia-induced obesity and higher plasma insulin levels^32^. The sixth ranked giDMR is in the *PRKCB* (protein kinase C, β) gene region, and another top giDMR is in the *PRKCB* TSS, and both are hypo-methylated in T2D cases. Increasing PRKCB levels caused by hyperglycaemia, are involved in insulin resistance^33^ and elevated PRKCB expression appears in the vasculature of T2D cases^34^.

### Whole-blood cell subtype analysis

The ideal tissue to study directly T2D pathophysiology would be the pancreatic β-cells; however, collection of this biological material is invasive and not feasible on a large scale. Peripheral blood is the best accessible alternative tissue that reflects multiple metabolic pathways. Early developmental epigenetic changes may be present in multiple tissues and additionally the T2D-associated metabolic syndrome is commonly associated with inflammatory processes^35^,^36^,^37^, which would be reflected in blood. Furthermore there is high potential clinical utility for any identified blood-derived epigenetic disease markers.

White blood cell (WBC) subtype proportions have been previously reported to associate with T2D^38^,^39^ and could possibly confound our associations. We find no significant association between estimated counts of lymphocytes, neutrophils, eosinophils and total blood cell counts with DNA methylation levels at the 4,545 (FDR 25%) T2D-DMRs. Only 2 out of the 4,545 DMRs are weakly associated with monocyte cell counts (chr21:34816251–34816750 linear regression *P*=0.01 and chr22:17742501–17743000 linear regression *P*=0.02). This suggests that differential blood cell types do not significantly confound the major T2D-methylation changes observed in our twin analysis.

### Medication use

To explore possible effects of T2D medication on our findings, we first tested for association between methylation level and medication at time of blood sampling. We find that 2 (0.04%) of 4,545 T2D-DMR are associated with medication (FDR<0.05). We then divided cases into those on and off medication at the time of blood sampling as a covariate. After adjusting for medication, both of these T2D-DMRs are still nominally associated with T2D status (linear regression *P*=0.02), suggesting that medication is not a major confounder.

### Conclusion

We have identified potential T2D-related changes in a genome-wide analysis of the whole-blood DNA methylome. These are located predominantly around the TSS, but also show hypermethylation within gene regions. We find DMRs in the promoters of genes, such as *MALT1* and *GPR61*, where epigenetic modifications likely act as markers of the disease process, and where metabolomics data may highlight the chemical sequelae. Although our MZ twin design can increase power to detect unbiased biological changes, the relatively small effect sizes require further replication of the results in an external independent cohort. Our study shows the clear scientific and clinical potential of integrating epigenomics with other omics data for common complex diseases. This integrated approach has enabled us to characterize the epigenome, and identify key DNA methylation changes occurring in both T2D-susceptibility genes as well as genes altered by the disease progression itself.

## Methods

### Twins

All participants in the study provided written informed consent in accordance with the St Thomas’ Hospital local ethics research committee. Altogether, 27 pairs of MZ twins (including 17 pairs of T2D-discordant twins, 3 pairs of T2D-concordant twin and 7 pairs of healthy concordant twin, Supplementary Data 1) were selected in the discovery phase from the TwinsUK cohort^40^. Each participant’s information was collected by interview and questionnaire. Weight and height were taken on the day of visit and were used to calculate BMI. The zyogsity is determined by standard zygosity questionnaire and confirmed by genotyping (60%). We defined T2D status by the serum fasting glucose level test (≥7 mmol l^−1^) and/or self-report of T2D in the questionnaire data. The healthy twin in discordant twins was required to have a serum fasting glucose level test of ≤5 mmol l^−1^. The replication sample set was also selected from the TwinsUK cohort and comprised 42 unrelated T2D cases and 221 controls matched for age, BMI and sex.

Whole blood was collected at the interview and stored at −80 °C in EDTA tubes. DNA was extracted using the Nucleon Genomic DNA Extraction Kit BACC3 and stored at −20 °C in TE Buffer. We also obtained WBC subtype counts. WBC counts were derived from fluorescence activated cell sorting of peripheral blood. WBC subtype specific cell counts were calculated by multiplying the proportion of the WBC count of each cell type within the total WBC count (estimated in thousands of cells per ml), for four cell types in our sample set: neutrophils, eosinophils, monocytes and lymphocytes.

### MeDIP sequencing (MeDIP-seq)

All sample preparations and MeDIP sequencing were performed by the BGI-Shenzhen, Shenzhen, China. Extracted DNA was fragmented using a Covaris sonication system and sequencing libraries were prepared from 5-μg fragmented genomic DNA. End repair, <A > base addition and adaptor ligation steps were performed using Illumina’s Paired-End DNA Sample Prep kit. Adaptor-ligated DNA was immunoprecipitated by anti-5mC using a commercial antibody (Diagenode) and MeDIP products were validated by quantitative PCR. MeDIP DNA was purified with ZYMO DNA Clean & Concentrator-5 columns and amplified using adaptor-mediated PCR. DNA fragments between 200 and 500 bp in size were gel-excised, and the amplification quality and quantity were evaluated by Agilent BioAnalyzer analysis. The libraries were subjected to highly parallel 50-bp paired-end sequencing on the Illumina GAII platform. All sequencing data passed initial quality checks for base composition (no exclusions) using FASTQC (v0.10.0; ref. 41). For each individual, ~60 million reads were generated and mapped onto hg19 using Novoalign V2.07.11 (ref. 42). After removing duplicates, we filtered data using quality score Q10, paired-end read criteria and fragment insert-size distribution checks, which resulted in ~30 million unique reads confidently mapped onto the human genome. We quantified methylation levels using MEDIPs^13^ producing absolute methylation signals (AMS) and the mean relative methylation score (RPM) in each 500-bp bin (overlap of 250 bp) across the genome and these windows were used for the DMR analyses. The replication samples were profiled following the same procedure as the discovery set, except they were sequenced using single-end MeDIP-seq. The genetic effects on the DMRs and the effect of DMRs on T2D were calculated using the AMS in each DMR. MeDIP-seq data have been deposited in EMBL under the accession code E-MTAB-3051.

### DMR and giDMR discovery

DMRs and giDMRs were calculated using the MEDIPS RPM values. For T2D DMR estimation, the RPM values at each 500 bp bin were normalized to N (0, 1; standard normal distribution) before the analysis. Using a linear mixed effects model, we regressed methylation levels at each 500-bp window on fixed-effect terms, which included disease status (at the same time as the visit for the blood and DNA draw), BMI, age (for the blood and DNA withdraw), sex and random-effect terms denoting family structure. giDMRs were characterized among 34 (17 pairs) discordant monozygotic twins and *p* values were obtained from one-sample parametric *t*-test to assess whether the mean difference within twin pair for each DMR was significantly different from 0. Results from the discovery analyses are presented at a false discovery rate (FDR) of 25% (nominal *P*=1 × 10^−4^), estimated by Benjamini–Hochberg (95) *q* value^43^. The effect of DMR on T2D is calculated using a linear mixed effects model, we regressed AMS value for each DMR on fixed-effect terms, which included disease status, BMI, age, sex and random-effect terms denoting family structure.

To investigate the potential effect of medication use and cell subtype bias, we used records of any diabetes medication use and cell subtype counts data in the discovery samples. At each of the 4,545 suggestive T2D-DMRs we tested the effect of medication record and cell subtype data of DNA methylation levels. We fit a linear mixed effects model—BMI, age, sex, medication use (or cell subtype counts) were incorporated as fixed-effects and we included a random-effect term denoting family structure.

### Illumina human methylation 450K array

Illumina Human Methylation 450K array data were used to validate the top T2D DMR results. The Illumina 450K array methylation values are reported as *β*, which represent the ratio of array intensity signal obtained from the methylated beads over the sum of methylated and unmethylated bead signals^44^,^45^.

In the validation, we matched 20 probes from whole-blood Illumina 450K array for 45 individuals to the top 31 DMRs (Supplementary Data 7). When a probe overlapped with the DMR, we used it for validation; otherwise, we selected the nearest probe to the DMRs within 10 kb. The methylation values for each probe were normalized to N (0, 1) before the analysis. We fitted a linear mixed effects model regressing methylation levels at each probe on the disease status of the individuals and included fixed effects (age, sex, BMI, beadchip, BS conversion efficiency and BS-treated DNA input) and random-effects (family-structure).

We also tested the selected DMRs methylation profile in skeletal muscle biopsies from 24 T2D patients and 6 controls (non-T2D but obese individuals).

### Metabolomic data

Among the 54 MZ twin individuals, 36 had plasma and/or serum metabolites profiles. Altogether, 503 metabolites were measured using non-targeted technology gas chromatography–mass spectrometry and liquid chromatography–mass spectrometry^46^. Metabolites were reported as median normalized, correcting for the interday machine tuning effect by dividing each metabolite’s value by the median per run for the day. Then, the data were further standardized before the analyses. For the 36 MZ twins, the mean difference of metabolite levels within twin pair (affected–unaffected twin) was calculated. One-sample non-parametric test (Wilcoxon signed-rank test) was calculated to assess whether the mean difference for each metabolite was significantly different from 0. 50 metabolites nominally associated with DNA methylation (*P*<0.05) were then tested in the replication sample set. The meta-analysis of discovery (*n*=36 IDs) and replication (*n*=24 IDs) samples provided the overall *p* value for the association between metabolite levels and DNA methylation profiles.

### Gene expression profiles

Gene expression results in adipose, skin and lymphoblastoid cell line tissue was extracted for 590 subjects from MuTHER study^19^. Gene expression levels were measured using the Illumina expression array HumanHT-12 version 3. Each sample had three technical replicates and log2-transformed expression signals, which were quantile normalized, first across three replicates of each individual, and then secondly by quantile normalization across all individuals. We used the transformed normalized residuals of the log-transformed gene expression array signal in this analysis.

### Genotype data

Genotype data for the individuals in this study were obtained on a combination of Illumina platforms (HumanHap300, HumanHap610Q, 1M-Duo and 1.2MDuo 1M custom arrays). The genotypes were called with the Illuminus calling algorithm (maximum posterior probability of 0.95). Imputation was performed using the IMPUTE software package (v2) using two reference panels: P0 (HapMap2, rel 22, combined CEU) and P1 (610K+, including the combined HumanHap610K and 1M array). After imputation, SNPs were filtered for MAF of >5% and IMPUTE info value of >0.8 (ref. 19).

### Methylation QTL identification

*Cis* methylation QTL at DMRs was analysed using SNPs within 50 kb of the region. For each DMR, the methylation values were normalized to N (0, 1), and we then fitted a linear model, regressing the methylation levels on fixed-effect terms including genotype, age and gender. Multiple testing was corrected for by the Bonferroni correction. We further tested whether methylation acts as a mediator between genotype and phenotype. This was assessed using the causal inference test^8^,^31^. The genetic effect on DMRs was calculated using a linear mixed effects model, we regressed the RPM value for each DMR on fixed-effect terms, which included disease status, BMI, age, sex and random-effect terms denoting family structure.

*Trans* methylation QTLs at DMRs were analysed in the replication sample using 2.1M SNPs in the genome (*P*=2.4 × 10^−8^, FDR=5%). For each DMR, the methylation values were normalized to N (0, 1), and we performed association analyses by using linear regression implemented in PLINK^47^, assuming additive genetic effects, with adjustment for age and sex.

### Pathway analysis

Pathway analysis was performed using two methods: Cytoscape v2.83 (ref. 48) and GREAT^49^. We used DMR or giDMR annotated gene list for Cytoscape analysis with FDR <0.001. For GREAT, we analysed separately the T2D-DMR and giDMR regions and applied the regional-based binomial approach with the maximum distal extension reduced to from 1 Mb to 150 kb.

## Author contributions

T.D.S., S.L.J., J.T.B. and J.W. conceived the study. W.Y. and J.T.B. led the analyses. K.S., I.E. and T.F. contributed to a part of the analyses. K.W., Y.X., F.G., H.W., Y.X., H.L., C.L.H. and L.Y. preformed sample selection, sequencing and contributed experimental or technical support. A.K.L., M.J.B., A.M., P.D., S.L.J. and M.M. contributed materials, reagents, technical support and discussion. W.Y., C.G.B. and J.T.B. prepared the manuscript, with contributions from K.W., K.S., A.K.L., M.J.B., S.L.J., M.I.M. and T.D.S. All authors read and approved the manuscript. J.T.B., J.W. and T.D.S. jointly supervised this work.

## Additional information

**How to cite this article**: Yuan, W. *et al*. An integrated epigenomic analysis for type 2 diabetes susceptibility loci in monozygotic twins. *Nat. Commun*. 5:5719 doi: 10.1038/ncomms6719 (2014).

**Accession codes:** MeDIP-seq data have been deposited in EMBL under the accession code E-MTAB-3051.

## Supplementary Material

Supplementary Figure Supplementary Figure 1

Supplementary Data 1 Demography of studying twins

Supplementary Data 2 List of DMR which mapped to genes

Supplementary Data 3 31 DMR genes validation

Supplementary Data 4 Metabolites associate with MALT1 methylation (meta-analysis p value<0.0001 and discovery p value <0.05)

Supplementary Data 5 DMRs in T2D GWAS loci

Supplementary Data 6 T2D DMRs in other diseases genes

Supplementary Data 7 DMR in known imprinted genes

Supplementary Data 8 giDMR which mapped to genes

Supplementary Data 9 giDMR DNA methylation difference in blood case-controls replication samples

Supplementary Data 10 Pathway analysis for giDMR gene
