# Supplementary material for: An integrated epigenomic analysis for type 2 diabetes susceptibility loci in monozygotic twins
Source: Nat Commun. 2014 Dec 12;5:5719. doi: 10.1038/ncomms6719 (PMC4284644; doi:10.1038/ncomms6719)
Supplement: Supplementary Figure — 1 [file ncomms6719-s1.pdf]

Supplementary Figure 1

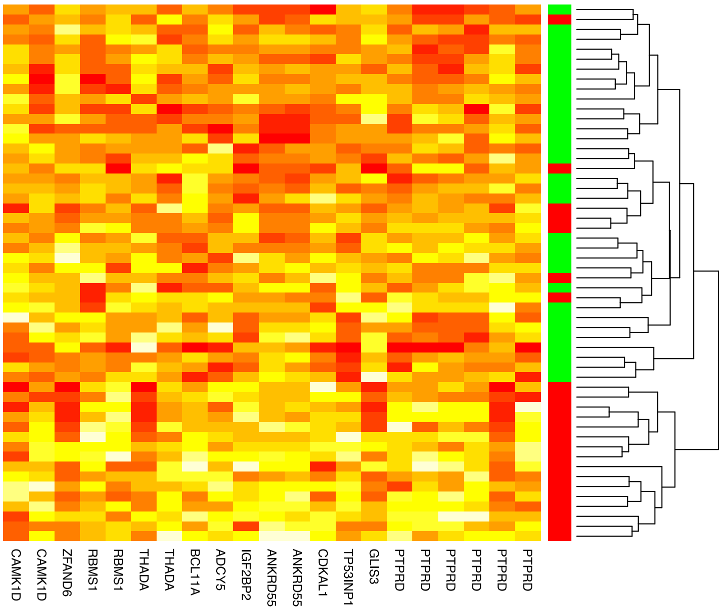

Hierarchical clustering of T2D DMRs and T2D giDMRs in T2D GWAS genes.
a. The heatmap indicates the level of DNA methylation at T2D DMRs ($p<0.0001$) (in red are hypermethylated DMRs, and in yellow are regions of hypomethylation). The DNA methylation-based hierarchical cluster on the right shows the participants (in red are T2D cases, and in green are healthy controls).

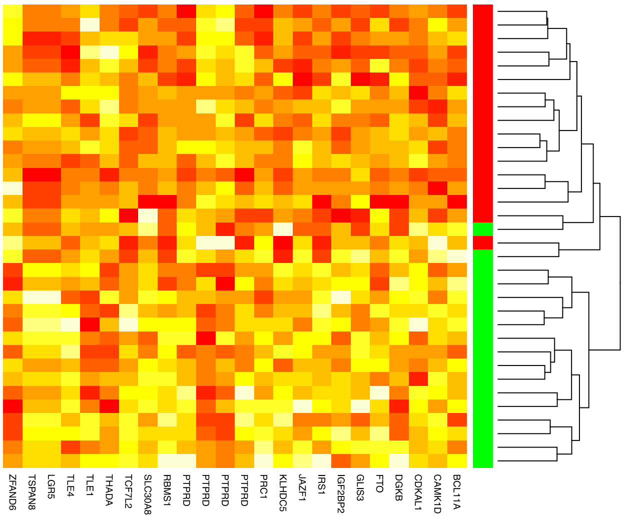

b. The heatmap indicates the level of DNA methylation at T2D giDMRs ($p<0.001$) (in red are hypermethylated DMRs, and in yellow are regions of hypomethylation). The DNA methylation-based hierarchical cluster on the right shows the participants (in red are T2D cases, and in green are healthy control).
